# Optimization of LCD-Based 3D Printing for the Development of Clotrimazole-Coated Microneedle Systems

**DOI:** 10.3390/ma18071580

**Published:** 2025-03-31

**Authors:** Oliwia Kordyl, Zuzanna Styrna, Monika Wojtyłko, Jolanta Dlugaszewska, Dorota Kaminska, Marek Murias, Dariusz T. Mlynarczyk, Barbara Jadach, Agnieszka Skotnicka, Bozena Michniak-Kohn, Tomasz Osmałek

**Affiliations:** 1 Chair and Department of Pharmaceutical Technology, 3D Printing Division, Poznan University of Medical Sciences, 3 Rokietnicka, 60-806 Poznań, Poland; sstyrnazuzanna@gmail.com (Z.S.); mwojtylko@ump.edu.pl (M.W.); 2 Chair and Department of Genetics and Pharmaceutical Microbiology, Poznan University of Medical Sciences, 3 Rokietnicka, 60-806 Poznań, Poland; jdlugasz@ump.edu.pl (J.D.); dorotakaminska@ump.edu.pl (D.K.); 3 Chair and Department of Toxicology, Poznan University of Medical Sciences, 3 Rokietnicka, 60-806 Poznań, Poland; marek.murias@ump.edu.pl; 4 Chair and Department of Chemical Technology of Drugs, Poznan University of Medical Sciences, 3 Rokietnicka, 60-806 Poznań, Poland; mlynarczykd@ump.edu.pl; 5 Chair and Department of Pharmaceutical Technology, Division of Industrial Pharmacy, Poznan University of Medical Sciences, 3 Rokietnicka, 60-806 Poznań, Poland; bajadach@ump.edu.pl; 6 Chair and Department of Pharmaceutical Technology, Poznan University of Medical Sciences, 3 Rokietnicka, 60-806 Poznań, Poland; agn.skotnicka@gmail.com; 7 Center for Dermal Research and Ernest Mario School of Pharmacy, Rutgers, The State University of New Jersey, Piscataway, NJ 08854, USA; michniak@pharmacy.rutgers.edu

**Keywords:** microneedle systems, additive manufacturing, 3D printing, fungal skin infections, clotrimazole

## Abstract

Fungal infections pose a significant global health problem, affecting 20–25% of the population and contributing to over 3.75 million deaths annually. Clotrimazole (CLO) is a widely used topical antifungal drug, but its efficacy is limited by poor penetration through the *stratum corneum*. Microneedle (MN) systems, composed of micron-scale structures arranged on a patch, offer a promising strategy to overcome the outermost skin barrier and enhance drug penetration into deeper layers. However, optimizing MN design, particularly in terms of size, shape, and fabrication technology, is essential for efficient drug delivery. This study aimed to develop CLO-coated MN systems using an Liquid Crystal Display (LCD)-based 3D printing technique and a thin-film dip-coating method. A comprehensive optimization of printing parameters, including anti-aliasing, layer thickness, curing time, and printing angle, was conducted to ensure the desired mechanical properties. The optimized MNs were coated with either suspension or ethanol-based CLO-hydrogels, with ethanol hydrogel demonstrating superior characteristics. Additionally, the study investigated how microneedle geometry and coating formulation influenced drug release. Antifungal activity against reference and clinical origin *Candida albicans* strains varied significantly depending on the coating formulation. Finally, the acute toxicity test confirmed no significant toxic effects on *Aliivibrio fischeri*, indicating the potential biocompatibility and safety of the developed MN-based drug delivery system.

## 1. Introduction

Due to the growing prevalence of microbial pathogens, fungal infections have become a significant global health burden. These conditions affect up to a quarter of the population worldwide [1,2,3]. Skin, hair, and nail infections are primarily caused by dermatophytes from the genera *Trichophyton*, *Epidermophyton*, and *Microsporum*. Additionally, *Candida* can be a pathogenic factor [3,4,5]. The global incidence of fungal infections differs depending on age, sex, or socio-economic and environmental conditions [5,6,7]. Dermatophytes exhibit optimal growth at surface temperatures ranging from 25 °C to 28 °C. Consequently, the prevalence of human skin infections increases in warm and humid environments, especially during the summer and rainy seasons in tropical and subtropical regions [2,5]. Moreover, the highest incidence rate is observed among elderly males [6]. Additionally, dermatophytic infections are more frequent in areas with lower socio-economic status due to overcrowded living conditions and poor hygiene [4]. Also, the population at risk of fungal infections continues to grow, driven by advances in modern medicine that have significantly extended the lifespan of individuals with previously incurable diseases [8]. Consequently, chemotherapy and radiotherapy following organ transplantation have increased the number of immunocompromised individuals susceptible to fungal infections [9]. It is worth noticing that the COVID-19 pandemic has further contributed to the spread of fungal co-infections [10]. Denning conducted a comprehensive analysis of fungal disease incidence between 2010 and 2023, revealing that over 6.5 million people suffered from fungal infections annually, leading to 3.75 million deaths. Notably, approximately 2.55 million of these fatalities were directly attributed to fungal diseases, without the presence of comorbidities [11]. The rising prevalence of fungal infections has triggered the increase in antifungal agents usage, subsequently accelerating the emergence of resistant strains.

Clotrimazole (CLO), an imidazole derivative, is one of the most extensively used antifungal agents. It belongs to the Biopharmaceutics Classification System (BCS) class II, characterized by low water solubility (0.49 μg/mL) [12] and good solubility in ethanol (20 mg/mL), chloroform or dimethyl sulfoxide [12,13]. CLO acts by inhibiting the cytochrome P450 14α-demethylase enzyme, thereby disrupting ergosterol biosynthesis, which is essential for fungal cell integrity and growth [14]. Although oral administration in the form of lozenges is licensed in certain countries, it is rarely given due to significant side effects, poor absorption, and significant systemic toxicity [15]. Standard antifungal management involves clotrimazole in 1% topical formulations such as creams, solutions, or lotions [16]. Nevertheless, the poor solubility of CLO in aqueous media, combined with the complex structure of the skin and unclear mechanisms of drug penetration, result in low topical bioavailability (~0.5%) [16,17]. The majority of the drug administered remains within the epidermis, particularly in the *stratum corneum* (SC), limiting the effectiveness and clinical outcome for infections located in deeper skin layers [16]. Furthermore, a topical formulation may cause adverse effects such as rash, hives, burning, itching, redness, swelling, or other forms of skin irritation [18]. Therefore, innovative drug delivery strategies are urgently needed to enhance the therapeutic efficacy of CLO while minimizing adverse effects and improving patient compliance.

Numerous advanced drug delivery systems have been investigated, including colloidal carriers (e.g., microemulsions and nanoemulsions) [19], vesicular carriers (e.g., liposomes, ufosomes, and niosomes) [20,21,22], nanoparticulate carriers (e.g., solid lipid nanoparticles (SLNs) and nanostructured lipid carriers (NLCs)) [23], or polymer-based systems (e.g., nanosponges, emulgels, and amphiphilic gels) [24]. To address the limitations of topical CLO delivery, Bolla et al. encapsulated CLO in ufosomes, resulting in significantly higher drug accumulation in the epidermis and dermis compared to conventional formulations [20]. Another study demonstrated the potential of CLO-loaded NLCs, where a CLO-NLC gel exhibited a four-fold increase in antimicrobial activity compared to both a conventional formulation and amphotericin B deoxycholate (served as a control drug) while maintaining a low toxicity profile [25].

Despite extensive research, scientific outcomes on new drug delivery systems remain limited. Hypodermic needles and cannulas have been crucial tools in medical practices for centuries. The concept of injecting substances under the skin dates back to the 17th century, when various devices were used, including glass tubes and quills [26]. With significant progress in medicine and pharmacy, there has been increased interest in improving systems for drug delivery through the skin. Consequently, innovative drug delivery methods, such as microneedle (MN) systems, have emerged as a promising alternative, enabling direct drug administration into deeper skin layers, which may enhance therapeutic efficacy. A pivotal advancement in microneedles (MNs) technology occurred in 1976 with the pioneering patent by Gerstel and Place, which laid the foundation for further research [27]. MN systems, composed of micron-scale needles (up to 2000 µm) arranged on a patch, allow for painless skin penetration, effectively overcoming the SC barrier and facilitating drug delivery into the epidermis or dermis [28,29]. In contrast to traditional hypodermic needles, microneedles create transient micropores in the skin without causing significant pain or bleeding. This characteristic renders them a more patient-friendly alternative for drug delivery. Despite initial concerns regarding potential risks, such as infection at the application site, studies suggest that microneedle-based administration results in lower microbial penetration compared to conventional hypodermic needles [30]. Furthermore, MN systems are being developed as self-administrable devices, offering patients greater independence and convenience. For comparison, the smallest needles commonly used for injections measure 30 G (gauge) (diameter: 0.31 mm, length: 13 mm), whereas 26 G needles (diameter: 0.45 mm, length: 13 mm) remain among the most frequently used. Coulman et al. performed a microscopic analysis of microneedle arrays with a needle height of 700 µm, comparing them to a 26 G needle tip. Their findings revealed considerable differences in dimensions, underscoring the potential benefits of MN systems for minimally invasive drug delivery [31]. MNs can be classified into various types, including solid, hollow, dissolving, coated, and swelling, each utilizing distinct drug delivery mechanisms [29,32]. Among them, coated MNs have gained attention due to their ability to incorporate an active pharmaceutical ingredient (API) within a thin hydrogel layer applied to the needle surface [33]. The coated MN delivery mechanism follows a “coat and poke” approach, where the drug dissolves upon skin penetration, allowing for diffusion into deeper layers [32]. However, the fabrication process of coated MNs requires precise optimization, particularly in selecting an appropriate hydrogel formulation and refining the coating process to accommodate the limited surface area of the microneedles.

Widely recognized for its success across diverse industries, additive manufacturing (AM), commonly referred to as three-dimensional (3D) printing, enables the precise fabrication of microneedle systems, allowing for accurate structures even with complex architectures. Furthermore, the customizable nature of 3D printing supports the adaptation of microneedles to individual patient’s needs, paving the way for personalized therapy. Several 3D printing technologies have been explored for MN systems fabrication, such as Fused Deposition Modeling (FDM) and vat photopolymerization techniques, including Stereolithography (SLA), Digital Light Processing (DLP), Two-Photon Polymerization (TPP), or LCD-based 3D printing [34,35]. SLA printing operates by layer-by-layer curing of photosensitive resin (serving as a building material) using an ultraviolet (UV) laser [36,37]. Fitaihi et al. demonstrated the capabilities of SLA 3D printing in fabricating micron-scale MNs with optimized geometric shapes for potential ocular applications, confirming their effective scleral and corneal tissue penetration [38]. While SLA technology is particularly promising due to its high precision and smooth surface objects, its primary drawbacks include high equipment costs and prolonged printing times [39]. On the other hand, DLP-based printing, utilizes a digital light projector to cure each entire layer simultaneously, significantly accelerating the printing process [36,37]. Reports indicate that DLP printing enables the fabrication of MN systems with sharp tips and sufficient mechanical strength [39]. However, while DLP offers increased printing speed, precision is reduced relative to the SLA technique. In contrast, FDM has been reported to be unsuitable for MN fabrication due to excessive stringing and visible geometric deformations [40]. Alternatively, Gittard et al. proposed TPP-based 3D printing as a high-resolution approach for MN production. The TPP process typically employs pulsed lasers with repetition rates in the tens of MHz, utilizing green (515–532 nm) or near-infrared light (~780, 1064, or 1080 nm) [41]. While this approach enables the fabrication of ultra-sharp microneedles, its major limitations include a very long production time and expensive, highly specialized equipment [42,43]. In recent years, LCD-based 3D printing has gained focus as a cost-efficient alternative between SLA and DLP technologies that balances precision and speed [39,44]. This technique employs UV light emitted by an array of light-emitting diodes (LEDs) passing through an LCD screen [34,45,46]. By reducing production costs, LCD printing enhances the accessibility of 3D-printed MN systems, making them more feasible for widespread medical applications [44,47]. Considering these advantages, Chanabodeechalermrung et al. proposed the use of LCD-based 3D printing for MN fabrication as an intermediate molding step in producing HPMC/PVP K90 dissolving microneedles for transdermal delivery of lidocaine hydrochloride (HCl) salt form (lidocaine HCl), demonstrating both safety and efficacy [48].

The selection of an appropriate 3D printing technology is a critical aspect influenced by multiple factors, particularly the intended application. Focusing specifically on vat polymerization techniques, additional key considerations include printer specifications such as resolution, print speed, printing material, and the cost of the printer (Table 1) [37,49]. These parameters often influence each other, leading to a complex interdependence that affects the overall process.

In 3D printing, resolution is defined in two dimensions: XY resolution and Z resolution. Z resolution refers to the minimum layer height, while XY resolution is determined by pixel size (for LCD-based and DLP printing), or by the laser beam spot size (for SLA and TPP printing) [50]. A higher resolution enables the fabrication of small-scale structures with intricate details. Reducing the layer thickness increases printing time, whereas thicker layers accelerate the process. Additionally, printing speed is influenced by the composition of the printing material [37]. Various combinations of monomers, oligomers, photoinitiators, and other additives determine resin properties and curing times. While most resins designed for LCD printers are also compatible with DLP printers, SLA- and TPP-specific resins differ due to variations in the light spectrum used for curing. Depending on the application, specialized resins are available, including high-strength formulations (e.g., ABS-like resins), high-speed resins, elastomeric photopolymers for flexible structures, or high-temperature-resistant resins [37]. Most commercial resins for TPP printing rely on acrylate- or epoxy-based chemistry [41]. It is worth noticing, that in medical applications, biocompatibility and resin safety are crucial considerations. The appropriate properties of printing materials can contribute to revolutionizing healthcare and regenerative medicine, enabling the fabrication of functional tissues, organs, and patient-specific implants [51,52,53,54]. Moreover, post-processing is an essential step to enhance the safety, mechanical properties, and stability of printed objects. This includes solvent washing (e.g., isopropyl alcohol), UV curing, and removal of support structures if they were added [55]. For TPP-based printing, additional post-processing techniques such as pyrolysis and isotropic plasma etching may be required, highlighting the complexity of the process [56].

In this study, we developed 3D-printed CLO-coated microneedles using an LCD-based 3D printing technique and a thin-film dip-coating method. Although numerous reports indicate the successful production of MN systems for drug delivery, a common challenge lies in achieving structures that are dimensionally and structurally consistent with the Computer Aided Design (CAD) specifications. Therefore, our study focuses on optimizing the design and printing parameters to maximize the printing quality and sharpness of the MN systems. Given the limited reports on precise parameter settings, this research will provide valuable insights for future studies. Our developed MN systems, characterized by sharp tips and appropriate mechanical properties, confirmed their potential capability for effective skin penetration. After coating with two types of CLO-loaded hydrogels—ethanol-based or suspension-based—we investigated drug release, antifungal properties, and potential toxicity of the CLO-coated MN systems. The results serve as a foundation for further research in the field of microneedle systems as a drug delivery platform.

## 2. Materials and Methods

### 2.1. Materials

Clotrimazole (Batch Number (B/N): 56NTH) was purchased from Pol-Aura (Poznan, Poland). Transparent photocurable resin (Anycubic^®^ Standard Clear, B/N: RPTT02418C0301) was obtained from Anycubic Corporation (Shenzhen, China). Carbopol^®^ EZ-3 (B/N: 0102679988) was provided by Lubrizol Corp. (Brussels, Belgium). Glycerol 85% (B/N: 094004) was purchased from Fagron (Olomouc, Czech Republic). Triizopropanolamine (B/N: MKCM7547) was sourced from Sigma-Aldrich (St. Luis, MI, USA). Additionally, 99.8% ethanol (B/N: 1317-03-24), isopropanol (B/N: 0808/10/15), acetonitrile (B/N: 1090-09-23), and potassium dihydrogen phosphate (B/N: 0008/01/22) were procured from Avantor Performance (Gliwice, Poland). Phosphate buffer concentrate (B/N: 220712042) and 96% ethanol (B/N:241022364) were purchased from Chempur (Piekary Śląskie, Poland). Agarose was supplied from Merck Chemical Company (Darmstadt, Germany), and 0.1% methylene blue was obtained from Aktyn (Suchy Las, Poland). *Candida albicans* ATCC 10231 reference strain was purchased from LGC Standards (Teddington, UK), and the clinical origin strain of *Candida albicans* was obtained from the University Hospital in Poznan (Poznan, Poland). Malt extract agar (B/N: VM1031598239) was purchased from Merck KGaA (Darmstadt, Germany). Microtox Acute Reagent (bacterium *Aliivibrio fischeri*), Microtox Diluent (2% NaCl), Microtox Osmotic Adjusting Solution (22% NaCl), and Microtox Reconstitution Solution were purchased from Tigret (Warsaw, Poland). Purified and deionized water was obtained from the Simplicity^®^ Water Purification System (Merck Millipore, Billerica, MA, USA).

### 2.2. Design of Microneedle Systems

**Figure 1:**
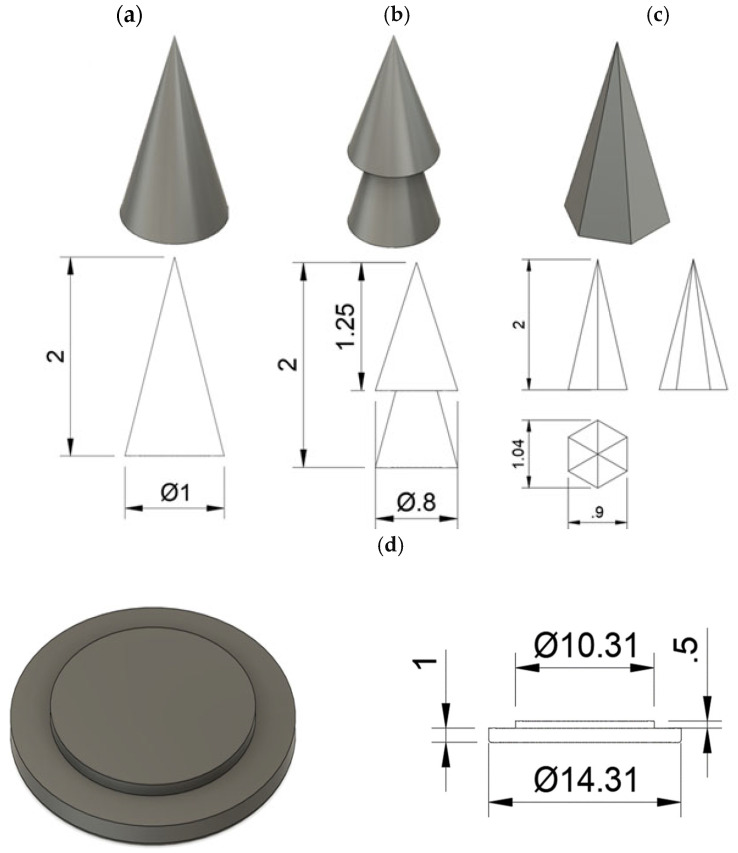
CAD model (upper) and 2D documentation (lower) of an individual microneedle with geometry: (**a**) cone (shape A); (**b**) double overlapping cone (shape B); (**c**) pyramid with hexagonal base (shape C); (**d**) the CAD design (left) and 2D documentation (right) of the double support base.

The microneedle systems were designed using AUTODESK^®^ FUSION 360^®^ software version 2601.0.90 (Autodesk Inc., San Rafael, CA, USA), resulting in three distinct needle geometries: a cone (shape A, Figure 1a), a double overlapping cone, in short DoC (shape B, Figure 1b), and a pyramid with a hexagonal base (shape C, Figure 1c). Each geometry was configured as an array containing either 17 or 37 individual needles, with a needle height of 2000 μm and inter-needle spacing of 2000 μm or 1330 μm, respectively. The base widths of the individual microneedles were designed as 0.80 mm for shape A, 1.00 mm for shape B, and 1.04 mm for shape C. To ensure structural integrity, the needles were mounted on a double-layered support base (Figure 1d), forming a fully integrated microneedle system. The CAD designs were exported as STL files and imported to the slicing software Photon Workshop V3.3.0 (Anycubic^®^, Shenzhen, China), which is responsible for converting the STL files into G-code/.pm3, the format used by 3D printers to create the physical objects layer by layer.

### 2.3. Manufacturing Process for Three-Dimensional (3D)-Printed Microneedle Systems

The microneedle systems were fabricated with an LCD-based 3D printer ANYCUBIC^®^ Photon Mono 5 (Anycubic Technology, Hong Kong, China). Transparent photocurable resin (Anycubic^®^ Standard Resin Clear) was used as the printing material. Following the 3D printing process, residual resin was removed by rinsing the MN systems with isopropanol in the ANYCUBIC^®^ Wash and Cure Machine 2.0 (Anycubic Technology, Hong Kong, China). Subsequently, microneedles were subjected to a 15-min curing process in a UV chamber ANYCUBIC^®^ Wash and Cure Machine 2.0 (Anycubic Technology, Hong Kong, China) at a wavelength of 385–405 nm to ensure complete photopolymerization.

#### 2.3.1. Optimizing Needle Density Within the Microneedle System

The optimization of needle density within the microneedle system was conducted by comparing two configurations: patches comprising either 17 or 37 microneedles. Both systems were fabricated using standard 3D printing parameters, which included a layer thickness of 0.05 mm, a curing time of 5 s, and a printing angle of 0°. The performance of both configurations was assessed by evaluating microneedle insertion efficiency using Parafilm^®^ M as a synthetic skin model. Parafilm^®^ M was cut into 10 squares, each measuring approximately 2 × 2 cm, and folded to create a multilayered model mimicking human skin. Each Parafilm^®^ M layer had a thickness of about 127 µm, resulting in a total thickness of 1.27 mm. Measurements were conducted using a Shimadzu AGS-X series texture analyzer (Shimadzu, Kyoto, Japan) and TrapeziumX software version 1.5.2 (Shimadzu, Japan). The MN systems were attached to the mobile probe of the texture analyzer using double-sided tape, while the Parafilm^®^ M was placed on the metallic base supported by a sheet of flexible foam. The probe was lowered onto the Parafilm^®^ M at a speed of 0.05 mm/s until a predefined force of 32 N was achieved, simulating the pressure exerted by a human thumb during the microneedle system application [57,58]. This force was sustained for 30 s, after which the system was lifted at a speed of 1 mm/s and subsequently removed. After completing the test, the Parafilm^®^ M layers were carefully separated, and the number of layers exhibiting full microneedle penetration was examined. Following this, a microscopic analysis of the MN systems was performed using a Nikon Eclipse TS100 microscope equipped with a DS camera (Nikon, Tokyo, Japan).

#### 2.3.2. Optimizing Three-Dimensional (3D) Printing Parameters

The optimal printing parameters were carefully established to ensure the quality and accuracy of the microneedle systems. Particular emphasis was given to four key parameters: anti-aliasing, layer thickness, curing time, and printing angle, as these factors critically influence the size, shape, and surface smoothness of the microneedles. Initially, the effect of anti-aliasing on 3D-printed MNs was assessed. Following this, microneedle systems were printed with varying layer thicknesses of 0.03, 0.05, and 0.1 mm for each design (curing time: 5 s). After determining the optimal layer thickness, the impact of different curing times (1, 3, 5, 7.5, and 10 s) on print quality was tested (printing on the build plate at 0°). Finally, the MN systems were printed at three different angles: 30°, 45°, and 60°. Following each parameter adjustment, the microneedle arrays were analyzed using a Nikon Eclipse TS100 microscope with a DS-SMc digital camera (Nikon, Tokyo, Japan). The needle length and/or base width were measured using Nikon NIS-Elements BR 3.0 software (Nikon, Tokyo, Japan) to determine the optimal settings.

### 2.4. Characterization of the Microneedle Systems

#### 2.4.1. Insertion Studies

The force required to pierce the skin depends on the microneedle dimension and geometry, with a proportional increase corresponding to the needle tip’s cross-sectional area [59]. The insertion depth of MN systems was assessed through insertion tests conducted on two different skin substitutes: Parafilm^®^ M and 2.65% agarose gel. The specific experimental setups for each substrate are described below.

##### Parafilm^®^ M Insertion

To evaluate the insertion performance of MNs, Parafilm^®^ M was employed, as described in Section 2.3.1. After completing the test, the Parafilm^®^ M layers were carefully separated, and the number of perforations in each layer was counted. The percentage of perforated areas was calculated using Equation (1):

(1) $$\%holes=\frac{the\;number\;of\;holes\;observed}{the\;number\;of\;microneedles\;}\;\times \;100$$

##### Agarose Gel Insertion

A 2.65% (*w*/*v*) agarose gel was prepared by dissolving 2.65 g of agarose in 100.0 mL of deionized water. The solution was heated in a microwave for 3 min until fully melted, then poured into a plastic container to a thickness of approximately 6 mm and left to solidify at room temperature for one hour. Measurements were conducted using a Shimadzu AGS-X texture analyzer (Shimadzu, Japan) and TrapeziumX software (Shimadzu, Japan). The MN systems were attached to the mobile probe with a double-sided tape, while the agarose gel in a plastic container was placed on the metallic base. The probe was lowered onto the agarose gel at a speed of 0.05 mm/s until a predefined force of 32 N was achieved. This force was sustained for 30 s, after which the system was lifted at a speed of 1 mm/s and removed. Following the removal of the MN system from the agarose gel, the cavities formed by the microneedles were stained with a 0.1% (*w*/*v*) methylene blue solution for 1 min to enhance the visibility of the insertion. The stained agarose gel was then excised and placed on a millimeter paper. The depth of the created cavities was captured using a camera and quantified with ImageJ software (version 1.54g).

#### 2.4.2. Mechanical Properties

The mechanical properties of the microneedle systems were evaluated using a Shimadzu AGS-X texture analyzer (Shimadzu, Japan) and TrapeziumX software (Shimadzu, Japan). Prior to testing, images of the microneedle systems were captured using a digital microscope (Nikon, Tokyo, Japan), and the initial needle height (*H*1) was measured. The MN systems were attached to the mobile probe with a double-sided tape and lowered onto the metallic base of the analyzer at a speed of 0.05 mm/s. A compressive force of 100 N was applied and maintained for 30 s to simulate mechanical stress, after which the system was lifted at a speed of 1 mm/s and removed. Following the compression test, the microneedle arrays were re-visualized, and the post-test needle height (*H*2) was measured. The reduction in needle height was calculated using the Equation (2) provided below:

(2) $$Variation\;in\;needle\;height\;(\%)=\frac{H1-H2}{H1}\times 100\%$$

### 2.5. Fabrication of Clotrimazole-Coated Microneedle Systems

#### 2.5.1. Preparation of the Clotrimazole-Based Hydrogels

Two 1% CLO-based hydrogels were prepared for microneedle coating: one containing CLO dissolved in 96% ethanol (CLO-EtOH) and another with a suspended drug (CLO-Sus). Glycerol and deionized water were added to a plastic container, followed by the addition of Carbopol^®^ EZ-3 on the surface. The mixture was stirred using a mechanical stirrer CAT R-50 (Ballrechten-Dottingen, Germany) at 600 rpm for 30 min. For the CLO-EtOH gel, CLO dissolved in ethanol was added 5 min before the end of mixing. Finally, a 40% triisopropanolamine solution was added dropwise as a neutralizing agent. For the CLO-Sus gel, CLO was incorporated into the final gel by grinding it in a mortar. Placebo gels (Placebo-EtOH and Placebo-Sus) were prepared using the same procedure, excluding the addition of clotrimazole. The compositions of the gels are presented in Table 2 [60,61].

#### 2.5.2. Coating Method for Microneedle Systems

**Figure 2:**
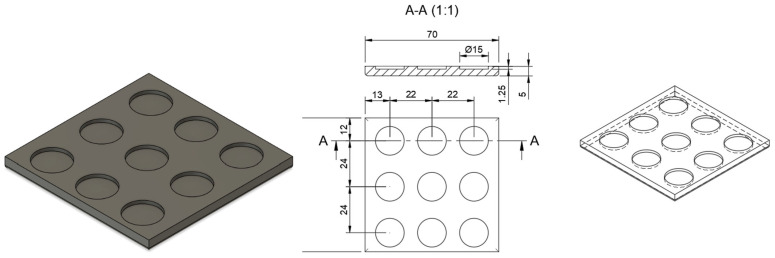
CAD model (**left**) and 2D documentation (**right**) of the coating plate.

Microneedle systems were individually coated with two types of hydrogels containing CLO, as well as placebo gels, using the thin-film dip-coating method [62]. This approach enables the precise and reproducible application of thin coatings facilitated by a shallow-depth reservoir. A custom-designed coating plate (Figure 2) was fabricated using photocurable resin (Anycubic^®^ Standard Clear Resin) and a 3D printer (Anycubic^®^ Photon Mono 5, Anycubic Technology, Hong Kong, China), employing the manufacturer’s standard 3D printing parameter. Each reservoir was characterized by a round-bottom structure with a height of 1250 μm, precisely engineered to achieve a coating of approximately two-thirds of the microneedles’ height. This configuration ensures selective coating of the microneedle tips while the base remains uncoated. The hydrogel was dispensed into the reservoirs using a spatula, and its surface was leveled while excess material was removed to ensure uniformity. The MN system was held with tweezers and gently immersed in the coating material by lowering it into the reservoir, followed by careful removal. The coated microneedles were left to dry at room temperature for a minimum of 8 h to facilitate the evaporation of volatile components. This process was repeated three times for each microneedle system to achieve consistent coating thickness.

### 2.6. Microscopic Analysis of Microneedle Systems

The structural characteristics of the microneedles, coated with either CLO-EtOH or CLO-Sus gel, were evaluated using a Keyence VHX-X1 Digital Microscope system (Keyence, Mechelen, Belgium), equipped with an automatic stage to enable a high-resolution (4K) analysis. Images were captured at magnifications ranging from 50× to 150×.

### 2.7. Pharmaceutical Evaluation of Clotrimazole (CLO)-Coated Microneedle Systems

#### 2.7.1. High-Performance Liquid Chromatography (HPLC) Analysis

The amount of clotrimazole was quantified using a validated method on High-Performance Liquid Chromatography (HPLC) apparatus (Shimadzu Nexera-I LC-2040C, Japan) and LabSolution Lite software version 5.82. A reversed-phase Kinetex^TM^ column with Phenyl-Hexyl 100, pores 2.6 μm, dimensions of 100 × 3 mm (Phenomenex, Torrance, CA, USA) was employed as the stationary phase, with the column temperature maintained at 40.0 °C. The mobile phase consisted of 25 mM potassium dihydrogen phosphate buffer pH 9.0 (45%) and acetonitrile (55%). The flow rate was 0.75 mL/min in isocratic mode. The UV detector was set at a wavelength of 210 nm, and the retention time for CLO was approximately 3.3 min.

#### 2.7.2. Drug Content Determination

To determine the drug content, a single microneedle system was placed in a glass container filled with 10 mL of ethanol. The sample was incubated for 1 h, with vortexing every 20 min to ensure complete dissolution of the drug. Afterward, the solution was diluted 2-fold with ethanol and analyzed using the HPLC method described in Section 2.7.1. The results were determined in triplicate to determine the mean value and standard deviation (SD).

#### 2.7.3. Evaluation of *In Vitro* Drug Release

Drug release studies were performed using Franz Diffusion cells (Teledyne Hanson Research, Chatsworth, CA, USA). The temperature was maintained at 32 ± 0.5 °C to reflect the skin temperature, with continuous stirring at 200 rpm. To maintain sink conditions, a phosphate buffer pH 7.4 with ethanol (70:30 *v*/*v*) was selected as the acceptor medium [60,63,64,65]. The microneedle system was directly applied to the orifice of the chamber (1 cm^2^ diameter) without the use of any membrane, allowing for direct contact between the microneedles and the medium. At predetermined intervals (6 min, 12 min, 30 min, 45 min, 1 h, 2 h, 3 h, 4 h, 6 h, 8 h, 10 h, 12 h), 0.4 mL of the acceptor fluid was withdrawn into HPLC vials and replaced with an equal volume of fresh medium to maintain the diffusion volume. An HPLC analysis was conducted as described in Section 2.7.1. The experiment was conducted in three replicates to determine the mean and standard deviation (SD).

### 2.8. Antifungal Properties

The antifungal activity of CLO-coated MN systems was assessed against standard and clinical origin strains of *Candida albicans* using the agar diffusion method. The inhibition zone was measured to determine the activity of clotrimazole delivered via the microneedle systems against *Candida albicans*. Both fungal strains were cultured on Malt extract agar (Merck KGaA, Darmstadt, Germany) under aerobic conditions for 24 h at 35 ± 1 °C. Following incubation, the cultures were resuspended in a 0.85% NaCl solution. The turbidity of the suspension was measured using a Grant-Bio DEN-1 Benchtop Densitometer (Riga, Latvia) and adjusted to a value of 0.5 McFarland units (corresponding to approximately 1.4 × 10^6^ CFU/1 mL for *C. albicans*; CFUs—Colony Forming Units). Then, a sterile cotton swab was dipped into the inoculum suspension and evenly spread over the entire surface of the agar plate by swabbing in three directions. MNs were applied manually to the agar plates with the needles facing downward. The study involved three different microneedle geometries (shape A, shape B, and shape C) coated with either CLO-EtOH or CLO-Sus gel. Drug-free MN systems coated with placebo-EtOH or placebo-Sus gels were used as negative controls. The plates were incubated at 36 ± 1 °C for 48 h. The inhibition zones were measured at 24 and 48 h using a ruler with an accuracy of 1 mm to evaluate fungal growth inhibition. All assays were determined in triplicate to evaluate the mean and standard deviation (SD).

### 2.9. Acute Toxicity

The acute toxicity of the microneedle system, whether coated with EtOH-CLO, Sus-CLO, placebo-EtOH, or placebo-Sus gel, as well as the uncoated system, was assessed using the 81.9% screening test using the Microtox M500 (Modern Water plc, Cambridge, UK) with Modern Water Microtox Omni 4.2 software [66]. *Aliivibrio fischeri*, a naturally bioluminescent bacterium emitting at a maximum wavelength of 490 nm, was used to evaluate toxicity [67]. Toxicity was determined by monitoring the bioluminescence of *A. fischeri*, which decreases upon exposure to toxic substances. Prior to the analysis, the bacteria were reconstituted and used within 5–6 h. To prepare the bacterial suspension, lyophilized bacteria (Microtox Acute Reagent, Modern Water plc, Cambridge, UK) were rehydrated with 1000 μL of Microtox Reconstitution Solution (Modern Water plc, Cambridge, UK) precooled to 5 °C, and further incubated at 5 °C for 15 min before being diluted with Microtox Diluent (150 µL of bacterial suspension and 1500 µL of Microtox Diluent) precooled to 15 °C. Such working bacterial suspension was added to each test cuvette (100 µL). The initial bioluminescence of *A. fischeri* (l_0_) was recorded. Subsequently, 900 μL of Microtox Diluent, a 2% sodium chloride solution (Modern Water plc, Cambridge, UK) precooled to 15 °C, was added to the cuvettes. Immediately thereafter, 17 individual microneedles from one system, previously excised using sterile tweezers, were introduced into the suspension. Considering that all microneedle shapes were fabricated using the same procedure, the analysis was limited to shape A (cone). Bioluminescence was measured at 5 and 15 min following the addition of the microneedles to assess any potential toxic effects. The experiment was performed in triplicate to determine the mean and standard deviation (SD).

### 2.10. Statistical Analysis

All statistical analysis was performed using Statistica software version 14.1.0.4, Cloud Software Group, Inc. 2023 (Fort Lauderdale, FL, USA); TIBCO software: https://www.tibco.com/ (Palo Alto, CA, USA), (accessed on 27 November 2024). Data were presented as the mean ± standard deviation (SD) from at least three independent repetitions. Statistical significance was assessed using a *t*-test and *p* < 0.05 was considered statistically significant.

## 3. Results

### 3.1. Optimizing the Manufacturing Process for Three-Dimensional (3D)-Printed Microneedle Systems

#### 3.1.1. Needle Density

**Figure 3:**
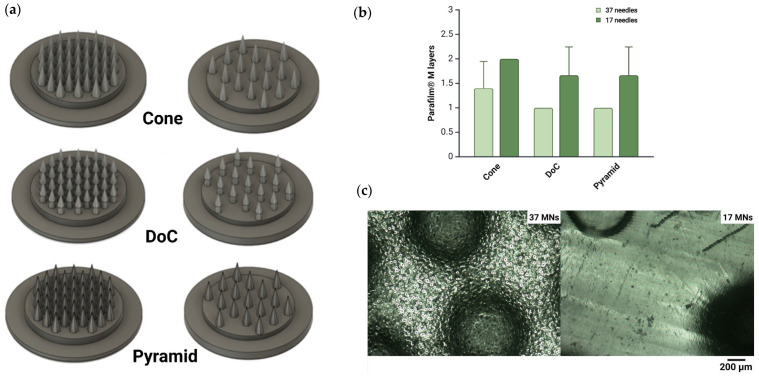
(**a**) The left panel illustrates the CAD models of MN systems containing 37 needles per patch, while the right panel shows systems with 17 needles per patch; (**b**) a comparison of penetration efficiency between the 17-needle and 37-needle MN systems. Data are presented as mean ± SD (*n* = 3); (**c**) microscopic analysis of the MN systems with 37 and 17 needles per patch.

Following the design of three distinct geometries of MNs—shape A (cone), shape B (double overlapping cone, DoC), and shape C (pyramid with a hexagonal base)—the optimal number of needles per patch was evaluated by comparing patches containing 37 and 17 microneedles (Figure 3a). The results indicated that patches with 17 microneedles exhibited significantly improved penetration efficiency, with increases of 33.34% for shape A and 40.00% for shapes B and C, compared to patches with 37 microneedles (Figure 3b). Additionally, a microscopic analysis revealed that the close spacing of needles in the 37-microneedle patches hindered the complete removal of residual resin after the 3D printing process (Figure 3c). Based on these findings, the 17-needle systems configuration was selected for further optimization steps.

#### 3.1.2. Anti-Aliasing

**Figure 4:**
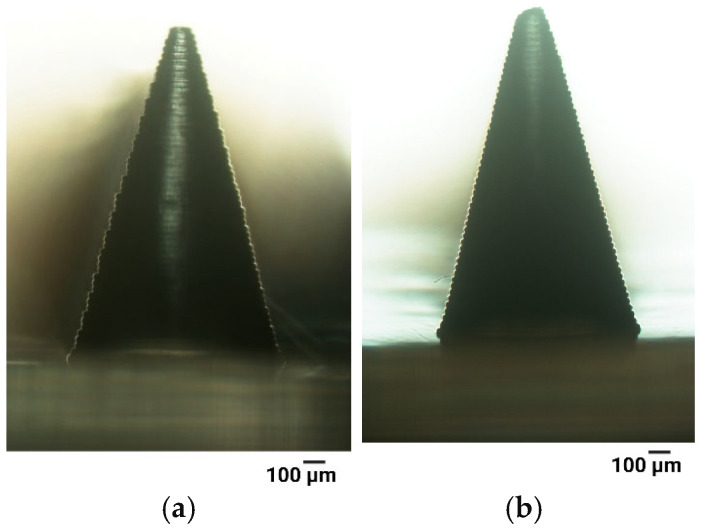
Representative microscopic images of cone-shaped microneedles: (**a**) without anti-aliasing; (**b**) with anti-aliasing.

Aliasing introduces defects such as uneven and stepped edges, posing a significant challenge during 3D printing [68]. To improve the resolution of 3D-printed microneedle systems, anti-aliasing was employed during the slicing procedure (Section 2.2). Figure 4a demonstrates that the microneedle printed without anti-aliasing exhibits a stepped surface, whereas this effect was reduced when the parameter was applied (Figure 4b). This effect was observed for all microneedle shapes, with the cone shape selected as a representative example to demonstrate the impact of anti-aliasing on the printing outcome. Therefore, the application of anti-aliasing was maintained for use in subsequent optimization stages.

#### 3.1.3. Layer Thickness

**Figure 5:**
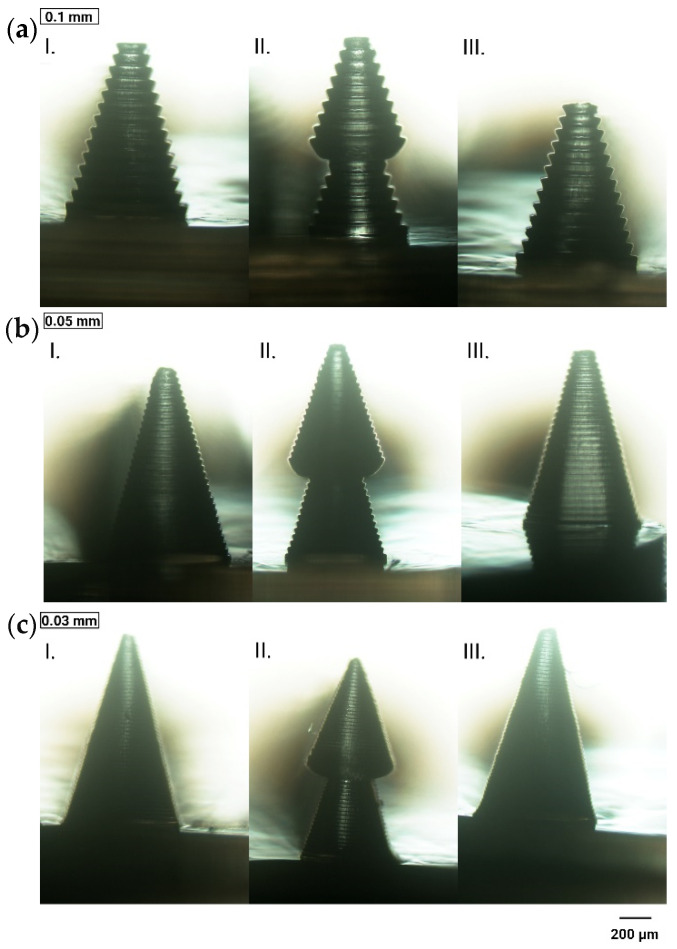
Microscopic images of MN systems (I—shape A; II—shape B; III—shape C) fabricated with varying layer thicknesses: (**a**) 0.1 mm; (**b**) 0.05 mm; (**c**) 0.03 mm.

Layer thickness directly correlates with the number of layers required for printing, affecting the overall printing duration. Three layer thicknesses of 0.1, 0.05, and 0.03 mm were evaluated to identify the optimal setting. As shown in Figure 5a, microneedles printed with a 0.1 mm layer thickness exhibited a stepped surface. This effect was reduced at a layer thickness of 0.05 mm (Figure 5b), with the smoothest surface achieved at 0.03 mm (Figure 5c). Moreover, Figure 5c reveals a sharper needle tip at the 0.03 mm layer thickness compared to the other configurations. Additionally, as the layer thickness decreased, the microneedle height aligned more closely with the specifications of the CAD design in most cases (Table 3). Accordingly, a layer thickness of 0.03 mm was selected for further optimization.

#### 3.1.4. Curing Time

Curing time, defined as the duration of UV light exposure required to polymerize liquid resin, is a critical parameter in 3D printing that significantly influences the dimensions and mechanical properties of fabricated structures [69]. To investigate its influence on the fabrication process, microneedles were printed using curing times of 1, 3, 5, 7.5, and 10 s with the printing angle fixed at 0°. Key dimensional parameters, including microneedle height and base width, were measured (Table 4). A curing time of 1 s was insufficient for all geometries, resulting in failed prints. At 3 s, geometries A and C were successfully printed with considerable deviations from the intended dimensions, while geometry B remained incomplete. Extended curing times of 5, 7.5, and 10 s allowed for the successful printing of all geometries. However, dimensional accuracy achieved after 5 and 10 s of exposure was lower compared to prints produced with a curing time of 7.5 s. While 7.5 s provided the most consistent results with the smallest standard variations, the dimensions continued to deviate from the CAD specifications. Therefore, further optimization efforts were directed toward refining the printing angle parameter to improve overall accuracy and achieve closer alignment with the target dimensions.

#### 3.1.5. Printing Angle

**Figure 6:**
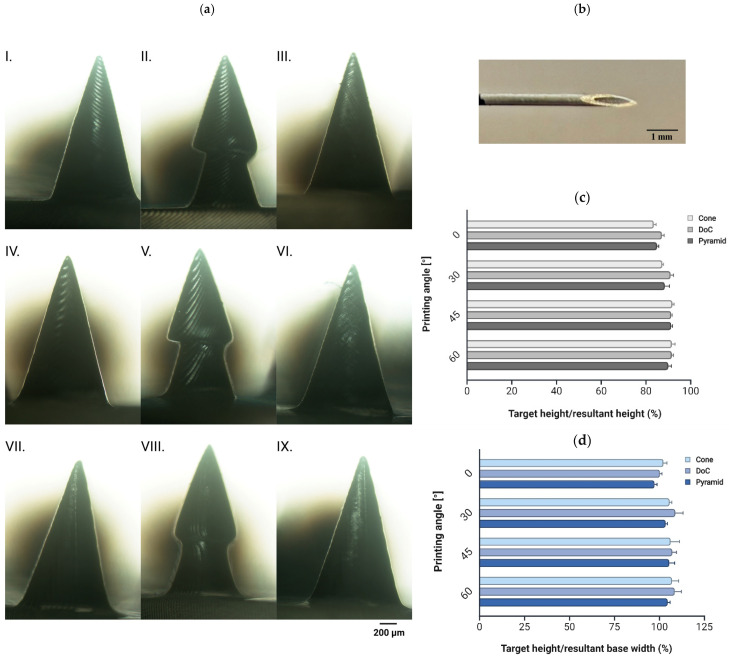
(**a**) Microscopic images of microneedles with shapes A (I, IV, VII), B (II, V, VIII), C (III, VI, IX) fabricated at printing angles of 30° (I–III), 45° (IV–VI), and 60° (VII–IX); (**b**) conventional hypodermic needle (16G); (**c**) accuracy of MNs height (target/resultant %); (**d**) accuracy of MNs base width (target/resultant %). Data are presented as mean ± SD (*n* = 3).

To evaluate the impact of the printing angle on the 3D printing process, microneedle systems were fabricated at orientation angles of 30°, 45°, and 60° on the -x and -y axes. Support structures were employed during printing to ensure stability and were removed post-fabrication. A microscopic analysis showed that printing at each tested tilted angle resulted in MNs with sharper needle tips (Figure 6a) than those printed at 0° (Figure 5c), making them a promising alternative to conventional hypodermic needles (Figure 6b). Furthermore, the printing angle influenced the dimensions of the fabricated MNs. Measured heights and base widths were compared to the target CAD specifications, with dimensional deviations expressed as percentages to assess printing accuracy. Among all tested orientations, printing at 30° resulted in the highest deviations for all shapes. For shapes A and C, the optimal printing angle was 45° (Figure 6a: IV, VI), achieving height deviations of approximately 8.18% and 8.73%, respectively, representing the closest alignment with CAD specifications (Figure 6c). Conversely, for shape B, all tested angles yielded similar needle heights. However, at 30° and 45°, individual microneedles exhibited visible deformation and lateral tilting (Figure 6a: II, V). In contrast, printing at a 60° angle produced structurally accurate needles for shape B (Figure 6a: VIII), with a height deviation of 8.45% (Figure 6c). A statistical analysis confirmed significant differences between printing at a tilted angle and printing at 0°, indicating a substantial improvement in needle sharpness and overall print quality. However, the base width increased across all tested angles, with deviations of 6.23% (shape A), 8.54% (shape B), and 5.48% (shape C) (Figure 6d).

Based on the comprehensive analysis of 3D printing parameters, the following configurations were selected for final fabrication and subsequent studies:

- Shape A (cone) and shape C (pyramid): 17 microneedles per patch, 0.03 mm layer thickness, 7.5 s curing time, 45° printing angle, with anti-aliasing applied;
- Shape B (DoC): 17 microneedles per patch, 0.03 mm layer thickness, 7.5 s curing time, 60° printing angle, with anti-aliasing applied.

### 3.2. Characterization of the Microneedle Systems

#### 3.2.1. Insertion Studies

##### Parafilm^®^ M Insertion

**Figure 7:**
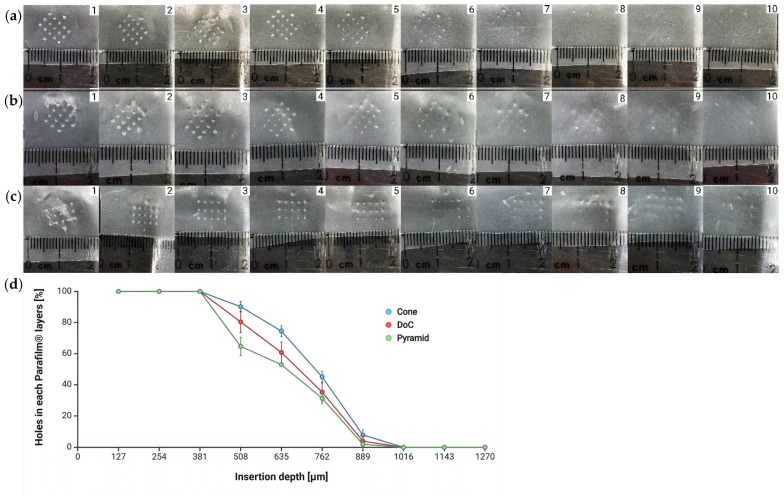
Representative images of perforations created by each microneedle geometry in successive Parafilm^®^ M layers: (**a**) shape A, (**b**) shape B, and (**c**) shape C; (**d**) the percentage of perforations achieved across individual layers for each microneedle shape. Numbers 1–10 sequentially represent the layers of Parafilm^®^ M. Data are presented as mean ± SD (*n* = 3).

Parafilm^®^ M has been proposed as a model that simulates the mechanical properties of porcine skin, which is similar in barrier properties to human skin [57,58]. Figure 7a–d demonstrates the successful full penetration of each microneedle shape through three Parafilm^®^ M layers (total thickness: 381 µm). No microneedles penetrated beyond the 8–10^th^ layers. Furthermore, the microneedle geometry did not influence the appearance of the perforations formed in the Parafilm^®^ M layers (Figure 7a–c).

##### Agarose Insertion

**Figure 8:**
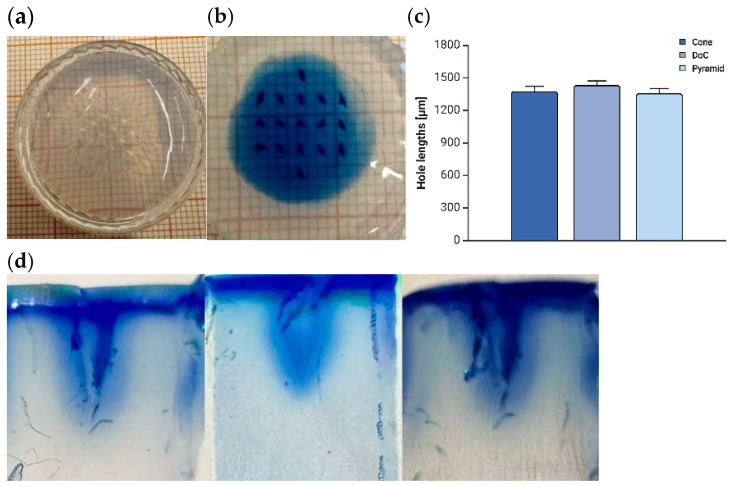
Representative images of perforations created by each microneedle geometry in 2.65% agarose gel: (**a**) immediately after insertion and removal of the microneedles from the gel, (**b**) after staining with 0.1% methylene blue; (**c**) hole lengths created by an individual microneedle inserted into agarose gel after staining with methylene blue. Data are presented as mean ± SD (*n* = 10); (**d**) individual microneedles inserted into agarose gel and stained with methylene blue, from left: shape A, shape B, and shape C.

Immediately after insertion, the perforations created by the microneedles were not visible (Figure 8a). However, they became apparent upon staining with 0.1% methylene blue (Figure 8b). Microneedles of all geometries successfully penetrated the agarose gel, creating holes that reflected their original needle shapes, with measured penetration depths of 1377.61 ± 41.92 µm, 1433.50 ± 38.47 µm, and 1352.98 ± 43.76 µm for shapes A, B, and C, respectively (Figure 8c,d).

#### 3.2.2. Mechanical Properties

**Figure 9:**
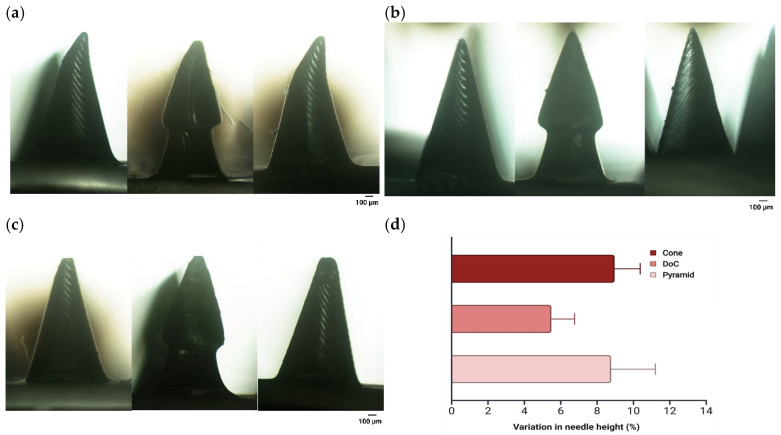
Microscopic images of microneedles (from left: shape A, shape B, shape C) after mechanical testing: (**a**) bent, (**b**) intact, (**c**) with reduced height; (**d**) variation in needle height (%). Data are presented as mean  ±  SD (*n*  =  3).

Mechanical testing revealed three distinct responses among all microneedle geometries when subjected to a force of 100 N for 30 s. Some microneedles demonstrated bending rather than fracturing (Figure 9a), while others maintained their structural integrity, exhibiting no visible deformation (Figure 9b). In certain cases, a reduction in microneedle height was observed, with decreases of 8.96%, 5.49%, and 8.77% for shapes A, B, and C, respectively (Figure 9c,d). For shape B, the lowest percentage reduction in individual microneedle height was observed, demonstrating a statistically significant difference compared to shape A, but not to shape C.

### 3.3. Microscopic Analysis of Microneedle Systems

**Figure 10:**
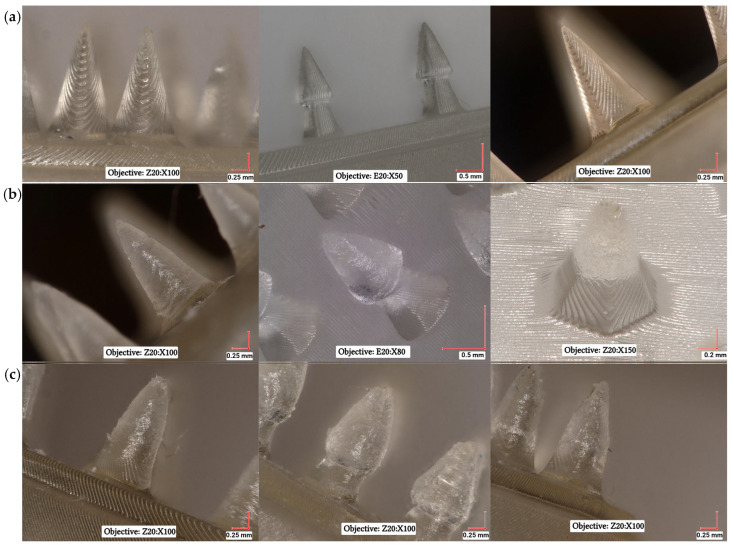
Digital microscopic images of microneedles with shapes A, B, and C, respectively: (**a**) before coating; (**b**) after coating with CLO-EtOH gel; (**c**) after coating with CLO-Sus gel.

To evaluate the impact of coating processes using CLO-EtOH and CLO-Sus gel on the surface coverage of microneedles, a microscopic analysis was conducted. Figure 10 presents the three microneedle geometries: (a) before coating, (b) after coating with CLO-EtOH gel, and (c) after coating with CLO-Sus gel. Microneedles coated with the EtOH-based gel exhibited a uniform and smooth coating layer, covering approximately two-thirds of the microneedle height, in accordance with the intended coating approach. In contrast, microneedles coated with the CLO-Sus gel displayed uneven surface coverage, characterized by irregular gel protrusions. This variability resulted in inconsistent drug loading across individual microneedles. Furthermore, the suspension gel formed a thicker coating layer, leading to the deformation of the microneedle geometry.

### 3.4. Pharmaceutical Evaluation of Clotrimazole (CLO)-Coated Microneedle Systems

#### 3.4.1. Drug Content Determination

Each microneedle system was subjected to extraction in ethanol, selected due to its excellent solubility of CLO, ensuring complete dissolution of the drug. Table 5 demonstrates that the extracted drug content for each microneedle shape was higher when coated with the CLO-Sus gel compared to the CLO-EtOH gel.

#### 3.4.2. Evaluation of *In Vitro* Drug Release from Microneedle (MN) Systems

**Figure 11:**
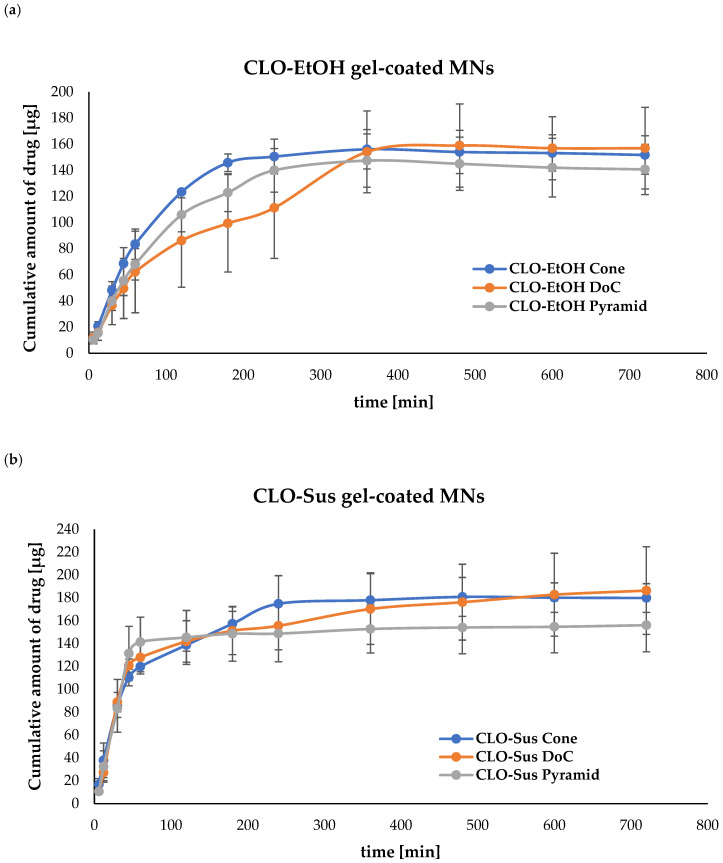
The cumulative amount of drug released during 720 min for microneedle shapes—cone (blue), double overlapping cone (orange), and pyramid (gray)—coated with: (**a**) CLO-EtOH gel; (**b**) CLO-Sus gel. Data are presented as mean ± SD (*n* = 3).

Figure 11a,b illustrates the release profiles of clotrimazole from microneedle systems with different geometries coated with either CLO-EtOH or CLO-Sus gel. The acceptor fluid was supplemented with ethanol to maintain sink conditions, addressing the limited aqueous solubility of clotrimazole. The type of coating gel did not significantly influence the total drug release after 12 h. For MNs coated with CLO-EtOH gel, the total drug release were 151.71 ± 14.73 µg, 156.87 ± 31.24 µg, and 140.64 ± 19.26 µg for shapes A, B, and C, respectively, whereas for CLO-Sus gel, the respective values were 179.80 ± 12.55 µg, 186.29 ± 38.33 µg, and 156.01 ± 23.29 µg. Notably, the MNs coated with the CLO-Sus gel exhibited drug release of 66.61% (shape A), 68.83% (shape B), and 90.56% (shape C) within the first 60 min, followed by either a gradual release or a plateau phase, depending on the geometry of the MNs (Figure 11b). In contrast, the MNs coated with the CLO-EtOH gel demonstrated a lower drug release, reaching 54.49% (shape A), 39.65% (shape B), and 48.38% (shape C) of the total CLO, within the same timeframe (Figure 11a). Furthermore, the release profiles varied based on microneedle geometry. Specifically, while geometries A and C exhibited similar release behaviors, geometry B demonstrated a two-stage release profile. A burst release phase was observed between 240 and 360 min for MNs coated with CLO-EtOH gel, with the drug amount increasing from 111.28 µg to 154.05 µg. For CLO-Sus gel-coated MNs, the burst phase was observed between 240 and 480 min. However, the increase in drug release was lower compared to CLO-Sus gel-coated MNs, rising from 155.48 µg to 176.15 µg.

### 3.5. Antifungal Properties

**Figure 12:**
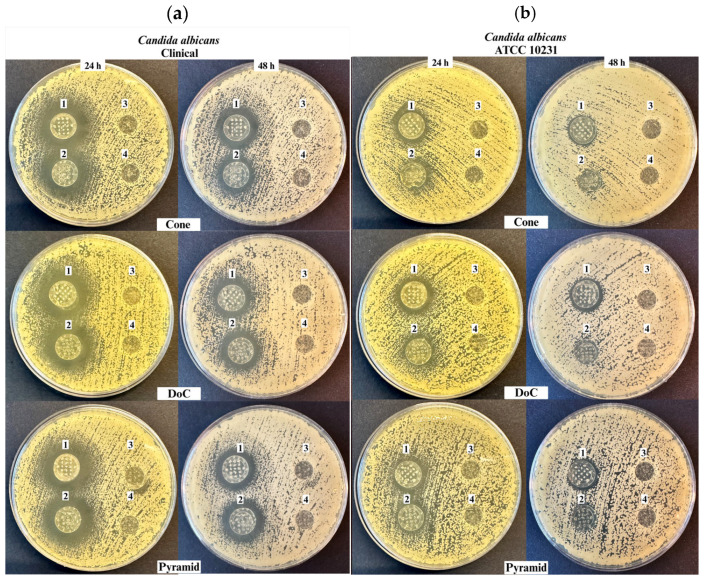

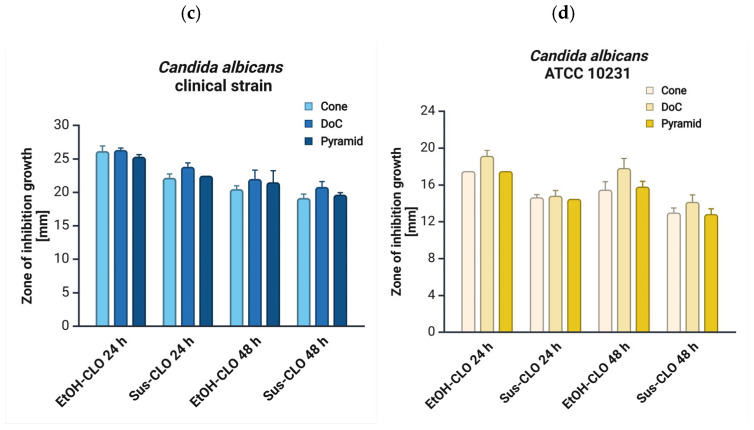
Representative images documenting the application of microneedle systems on agar plates inoculated with: (**a**) *Candida albicans* clinical origin strain, (**b**) *Candida albicans* ATCC 10231 reference strain; (**c**) zone of inhibition of growth for CLO-coated microneedle systems against *Candida albicans* clinical origin strain after 24 and 48 h of incubation; (**d**) zone of growth inhibition for CLO-coated microneedle systems against *Candida albicans* ATCC 10231 after 24 and 48 h of incubation (1—MNs coated with CLO-EtOH gel; 2—MNs coated with CLO-Sus gel; 3—MNs coated with placebo-EtOH gel; 4—MNs coated with placebo-Sus gel). Data are presented as mean ± SD (*n* = 3).

As shown in Figure 12a–d, zones of growth inhibition were observed for microneedles coated with CLO-EtOH (labeled as 1 on plates) and CLO-Sus gels (labeled as 2 on plates), against both *Candida albicans* clinical origin strain and *Candida albicans* ATCC 10231 reference strain after 24 and 48 h of incubation. The type of coating gel significantly influenced the inhibitory effect, particularly at the 24 h time point, with CLO-EtOH-coated microneedles exhibiting larger inhibition zones against both strains compared to those coated with CLO-Sus gels, irrespective of microneedle shape. Notably, inhibition zones were larger against the clinical origin strain across all microneedle shapes, gel types, and time points. Throughout the entire incubation period, no zone of inhibition was observed for the microneedles coated with placebo-EtOH or placebo-Sus gel (labeled as 3 and 4, respectively).

### 3.6. Acute Toxicity

**Figure 13:**
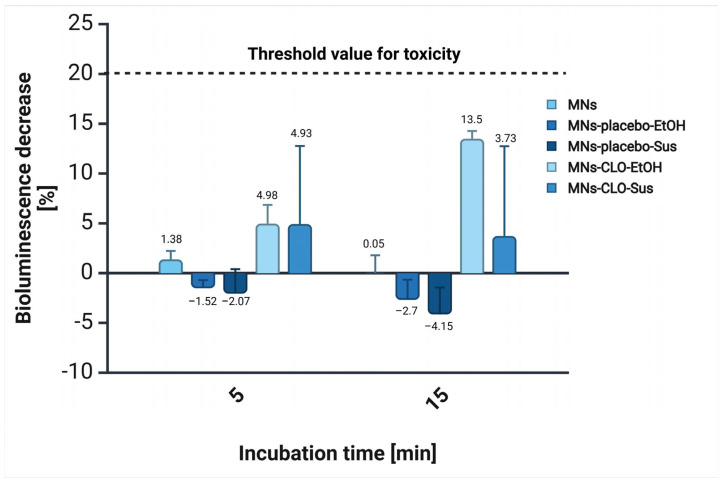
Changes in the bioluminescence of *Aliivibrio fischerii* after contact with various microneedle systems following 5 and 15 min.

Figure 13 presents the results of the acute toxicity assessment of various microneedle formulations, indicated by the changes in *Aliivibrio fischeri* bioluminescence. The arbitrary threshold is generally established at 20%, with values ≤20% indicating that the sample is considered non-toxic [70]. Uncoated microneedles exhibited minimal toxic effect at both 5 and 15 min, with a decline over time [71]. Interestingly, microneedles coated with placebo gels showed negative values in terms of bioluminescence reduction, suggesting a hermetic effect. On the other hand, microneedles coated with CLO-containing gels showed a low toxicity profile for both formulations. The MNs coated with CLO-EtOH gel led to a 4.98% bioluminescence decrease after 5 min, with a further increase of up to 13.5% at 15 min. In contrast, MNs coated with Sus-CLO gel showed a similar decline in the emitted light at 5 min, however, it decreased to 3.73% after 15 min. All observed values remained below the threshold, confirming that the tested samples were non-toxic to *A. fischeri*.

## 4. Discussion

In this study, we present an approach for formulating clotrimazole-coated microneedle systems using an LCD-based 3D printer, confirming their potential pharmacological efficacy. While 3D printing allowed for the successful fabrication of three distinct geometries—cone, double overlapping cone (DoC), and pyramid with a hexagonal base—resolution limitations necessitated the initial optimization of printing conditions.

It has been established that a circular supporting base exhibits superior insertion efficiency compared to a square shape [72]. Therefore, each system consisted of either 17 or 37 microneedles embedded in a circular supporting base. Initially, the analysis focused on optimizing the microneedle density within a single system, as this parameter directly impacts penetration efficiency [73]. While a higher microneedle count may seem advantageous, our study revealed that patches containing 17 MNs achieved more effective penetration of Parafilm^®^ M compared to those with 37 microneedles. This phenomenon is described by the “bed of nails” effect, indicating that smaller microneedle spacing requires additional force for effective insertion [74]. Similarly, Yan et al. demonstrated that microneedle arrays with lower needle densities were more effective in enhancing acyclovir flux compared to those with higher density [75]. Furthermore, numerous scientific reports highlight the critical importance of the post-printing stage in ensuring the quality and safety of printed microneedle patches [76,77]. Typically, the printed material consists of a photoinitiator, resin, and monomer. The presence of unreacted or residual monomers remains a critical concern, as their potential release into the skin may induce biological responses and adverse effects [78]. Therefore, the use of photocuring 3D printing techniques, including LCD-based 3D printing, for direct and prolonged contact with living tissues remains still under investigation in the academic area. Despite their numerous advantages, further advancements in fabrication methods and material development are essential to facilitate their broader application in biomedical engineering. Petrochenko et al. demonstrated the successful printing of supports, which were subsequently seeded with mesenchymal stem cells (MSCs) [79]. The photosensitive resin used in this work consisted of polyurethane diacrylate (UDA) resin (Genomer 4215), and two photosensitive diluents—2-hydroxyethyl acrylate and glycol diacrylate. The printed samples were repeatedly washed with organic solvents to remove unpolymerized resin and diluents. This further underscores the crucial role of the post-printing stage in ensuring the final quality of the printed structures, along with the comprehensive optimization of the manufacturing process. In our study, the microscopic analysis revealed that unwashed resin was observed in 37-needle patches post-printing, while no residue was detected in 17-needle patches.

Minimizing stair-stepping defects continues to be a key challenge, especially in LCD-based techniques, where the pixels are disconnected [80]. This issue has been previously highlighted in the literature as one of the limitations of 3D-printed MNs [38,43,80,81]. Each projected image is composed of pixels that can be either turned on (ON) or turned off (OFF). When a pixel is ON, the light source selectively exposes the resin, leading to localized curing, while OFF pixels block the light, preventing polymerization. Consequently, the printed surfaces exhibit a pixelated appearance under magnification. “Anti-aliasing” improves print resolution by introducing grayscale into otherwise black-and-white images [43]. Johnson et al. proposed a custom-optimized anti-aliasing algorithm using MatLab r2016b (Mathworks, Natick, MA, USA), enabling the precise modulation of light intensity across individual pixels [43]. In another study, a simpler approach was adopted by utilizing the automatic option provided by the slicer software [81]. To enhance the printing process, we utilized the anti-aliasing feature provided by the slicing software Photon Workshop, eliminating the need for time-consuming and complex procedures. This method significantly enhanced surface smoothness, which was further improved by optimizing layer thickness. Selecting a layer thickness of 0.03 mm increased the total number of layers, consequently extending the printing duration. On the other hand, using thicker layers reduced the total number of layers, accelerating the printing process, but as noted, led to the decrease in the resolution of the printed microneedles. However, other studies also report that extending the printing process can be more beneficial for achieving higher-quality prints, which is consistent with our observations and parameter selection [38,43,80,82].

The total time required to print a single layer comprises curing time, layer separation time, and resin refill time [83]. Among these, curing time directly influences the mechanical properties and dimensional accuracy of the printed structures. Additionally, the separation force exerted during layer detachment is strongly correlated with resin viscosity, with highly viscous resins presenting significant processing challenges [83]. Various photocurable resins used for the fabrication of microneedle systems have been described in the literature, including acrylic-based eResin PLA (viscosity: 200–300 mPa·s) [48], Autodesk Standard Clear PR48 Resin (viscosity: 400 mPa·s) [43], or resins from Anycubic manufacturer [84,85]. Moreover, a common choice is UV-curable resins provided by Formlabs Inc., Somerville, MA, USA [82,86,87,88]. However, these resins often exhibit high dynamic viscosity such as 900 mPa·s for Clear Resin V4, which may prolong the curing time and/or reduce the resolution of printed objects [82]. In this study, a transparent Anycubic^®^ Standard Resin with a relatively low viscosity of 200–230 mPa·s was selected as a printing material, ensuring excellent fluidity. Resin properties facilitate the fabrication of strong mechanical structures while maintaining high printing accuracy and fine detail resolution. It is worth mentioning, that during each layer separation, the build platform must move upward to detach the cured structure from the FEP (Fluorinated Ethylene Propylene). After polymerization, the cured layer adheres strongly to the transparent FEP, leading to potential deformation of the printed object when separation forces are applied [83]. Therefore, optimizing the curing time remains one of the key challenges during LCD-based 3D printing. In this study, a curing time of 7.5 s was determined to be optimal for all tested geometries. Notably, a 1 s exposure time resulted in complete print failure across all designs due to inadequate adhesion between the resin and the build platform, leading to the polymerized material adhering to the FEP foil. Consequently, an extended curing duration was necessary to ensure successful prints. Although a 5 s curing time allowed for the fabrication of structurally intact microneedle systems, deviations from target dimensions suggested still insufficient polymerization. Conversely, excessive exposure compromised mechanical integrity, likely due to over-polymerization, as observed with the 10 s curing condition. Interestingly, a curing time of 3 s resulted in incomplete printing for shape B. While the circular base was successfully printed, the individual microneedles exhibited structural deformation. This failure may be attributed to the complexity of the double-overlapping cone geometry, which likely caused unintended adhesion to the FEP film, resulting in defects.

Although printing directly on the build plate at a 0° angle enabled the successful development of different microneedle geometries, our observations confirmed previous reports in the literature indicating that dimensions of MNs deviated significantly from the target values, with blunt tips formation [43,89,90]. Johnson et al. explored stereolithographic 3D printing for MN fabrication and reported that the printed structures were approximately 30% shorter than the intended height of 1000 µm. To address this issue, they tested an approach in which CAD models were designed with heights of 1400–1500 µm, allowing the printing error to compensate for the reduction and achieve the desired final height of 1000 µm [43]. In another study, when using an LCD-based 3D printer, printing at a 0° angle resulted in a 20% reduction in microneedle height [39]. Our findings further demonstrated that the printing angle significantly affects the dimensions of microneedles, including height, base width, and tip sharpness, with optimal angles varying based on MN geometry. Consistent with the findings of Chanabodeechalermrung et al. [48], we determined that printing angles of 45° were optimal for shapes A and C, whereas shape B required an increased angle of 60°. The difference could be attributed to the smaller base diameter of shape B (0.8 mm) compared to shapes A (1 mm) and C (1.04 mm). As round-based MNs required greater material deposition, a higher angle was necessary to ensure structural integrity. Moreover, printing shape B at different angles resulted in structural deformation, causing the microneedle to tilt to one side. Although printing at an angle improved the quality of the printed structures, 8.18% (shape A), 8.45% (shape B), and 8.73% (shape C) reductions in microneedle height were still observed. These findings align with those of Jeong et al., who reported a 7.90% height reduction when printing MNs with a nominal height of 1000 µm at a 40° angle for subsequent polyimide microneedle molding [89]. Interestingly, other reports indicate that the height at a 0° orientation was almost consistent with the target dimension, while as the printing angle was tilted, the height increasingly deviated from the target [38,88]. The reduction in height was explained by insufficient structural support at certain points and gravitational effects during the 3D printing process. These variations highlight the specificity of different 3D printing technologies, as well as the influence of the chosen 3D printer and resin, underscoring the necessity for a comprehensive analysis in each study [90]. Additionally, when printing sharp structures such as microneedles, the uppermost layers are very thin and may not be properly cured. As a result, these layers may adhere to the FEP film rather than integrate with the structure, further contributing to height reduction. Based on these findings, we concluded that a reduction in final dimensions is a common phenomenon observed in microscale 3D printing [87]. On the other hand, different observations were noted regarding the base dimensions of individual microneedles. While printing at 0° resulted in a base diameter nearly identical to the CAD specifications, tilting the structure led to a slight increase in base dimensions. Similarly, Mathew et al. reported that during LCD-based 3D printing of microneedle systems, printing without tilting significantly reduced the microneedle height, whereas the base diameter remained consistent with the target dimensions [39]. Other studies have indicated that the base diameter of microneedles changed from a rounded shape at 0° to an elliptical form at 45°, becoming more elongated at 67.5° and 90°. When the initial base diameter was 450 µm, measurements showed only a 0.78% increase at 0°, but 17.37%, 19.72%, and 32.03% increases at 45°, 67.5°, and 90°, respectively, leading to significant deviations from the target dimensions [38]. Conversely, Choo et al. reported that printing at 45° or 60° did not significantly alter the base dimensions [88].

Since a sharp tip is crucial for an effective microneedle penetration of the stratum corneum [91], penetration capability was evaluated using Parafilm^®^ M [58] and a 2.65% agarose gel [92], as skin substitutes. Our findings demonstrated that all MN system shapes successfully penetrated three layers of Parafilm^®^ M (381 µm). The thickness of skin layers varies depending on anatomical location and age, while the epidermis ranges from 77 to 267 µm [93], with the stratum corneum measuring approximately 10 to 30 µm [94], and the dermis between 300 and 5000 µm [95]. These values confirm that the microneedles are capable of bypassing the stratum corneum and easily penetrating the skin layers. Previous studies have indicated that dissolving microneedles fully penetrated only a single Parafilm^®^ M layer [96], whereas other studies reported the penetration of two full layers after insertion [38,97,98]. This difference compared to our findings may be attributed to the lower mechanical strength of dissolving microneedles, which require a greater insertion force for successful penetration. A comprehensive analysis conducted by Zhang et al. demonstrated that a 2.65% agarose gel most accurately mimics porcine skin. Their study revealed that MNs with a length of 1500 µm penetrated approximately 76.6% of their total length, closely aligning with our results [92]. Another approach to assessing penetration in agarose gel involved measuring dye diffusion, where penetration into a 2.5% agarose gel was evaluated based on sulforhodamine B dye release from the microneedles [88].

The purpose of the mechanical testing was to assess the strength of the microneedles under forces higher than those expected during patient application. Various studies have employed different force levels, including 32 N, 100 N, or 300 N, to assess microneedles strength [39,97,99]. In this study, a force of 100 N was deemed sufficient to identify potential structural defects unrelated to skin application. The results showed that while some microneedles underwent crushing or bending, others remained structurally intact. Similar studies have confirmed that at 100 N, microneedles do not break entirely but undergo crushing, which is a crucial safety feature [99]. Furthermore, Mathew et al. reported that a force of 300 N resulted in microneedle bending rather than complete breakage [39]. The higher resistance to a 100 N compressive force in the case of shape B compared to shape A may be attributed to its complex structure, where two overlapping cones provide enhanced mechanical support under applied force. However, the mechanical strength test results did not correlate with the findings from the Parafilm^®^ M insertion study. Each microneedle shape successfully penetrated three layers of Parafilm^®^ M with 100% efficiency. Further analysis of individual layers revealed that the fourth layer was most effectively inserted by shape A, despite its lowest mechanical performance in compression tests. Observations of additional Parafilm^®^ M layers also indicated that the highest percentage of insertion was achieved with shape A. We conclude that since the microneedles did not completely fracture under a force approximately three times higher than those expected during patient-administered insertion, they will be safe under lower forces and will not remain in the skin.

Among various coating techniques—such as thin-film dip coating, immersion coating, drop coating, masked dip coating, spray coating, layer-by-layer coating, or roll coating [62]—thin-film dip coating is particularly advantageous for microneedle applications due to its simplicity and ability to precisely coat complex geometries [100,101]. Chen et al. proposed a dip-coating process utilizing an adjustable apparatus capable of controlled lifting and lowering. By lowering the portable holder with attached MNs, the coating layer was applied to their surface, allowing for precise and uniform coating [102]. In the present study, a thin film was prepared using a shallow reservoir with a depth smaller than the length of the microneedle systems, a method previously reported in the literature, that enabled successful coating [62]. Our previous studies indicated that both gel formulations—containing the drug either in dissolved (EtOH-gel) or suspended form (Sus-gel)—could be potentially applied to the skin [60,61]. Therefore, microneedle systems were coated with both types of CLO-containing gels. The microscopic analysis revealed that the CLO-Sus gel formed an uneven coating on the microneedles, which may influence the drug dosage applied, leading to reduced reproducibility and, consequently, variations in drug release, as well as skin penetration. In contrast, the CLO-EtOH gel provided a more uniform and consistent coating. Our previous studies utilizing the same gel compositions with a different active compound, agomelatine, demonstrated that the EtOH-based gel exhibited lower hardness, higher cohesiveness, and better spreadability [61]. Since the addition of the active ingredient did not significantly alter the formulation properties, and the placebo gels behaved similarly, these findings may correspond with the results obtained in our study. Based on these observations, the EtOH-CLO gel appears to be more suitable for microneedle coating due to its more uniform application and better formulation characteristics.

Drug content determination confirmed the above conclusions, showing that microneedles of all shapes coated with the CLO-Sus gel contained higher amounts of drug compared to those coated with the EtOH-based gel, despite utilizing the same coating method. Considering the total volume of the single microneedle, which was 0.52, 0.43, and 0.47 mm^3^ for shapes A, B, and C, respectively, it was expected that the cone-shaped microneedles would have the highest drug load. Indeed, the highest CLO content was observed in shape A, but only when coated with the CLO-Sus gel. This may be attributed to the previously mentioned uneven coating that occurs when the drug is in a suspended form.

The drug release study demonstrated that MNs coated with CLO-Sus gel exhibited a different drug release profile compared to those coated with CLO-EtOH gel, showing a faster initial drug release. However, it was expected that the formulation in which the clotrimazole is dissolved would release the drug faster than the formulation where the drug remains suspended. This phenomenon may account for the rapid release of the drug from the gel, which formed an irregular coating extending beyond the microneedle surface, while the provided sink conditions facilitated the dissolution of the suspended drug. The protruding particles dissolve immediately upon contact with the acceptor fluid, whereas in the case of CLO-EtOH gel, the smooth three-layer gel coating on each microneedle may contribute to the gradual release of clotrimazole. In our previous studies, we also reported similar observations, where the burst release for MNs coated with CLO-Sus gel occurred earlier than for those coated with CLO-EtOH gel [60]. Consequently, the suspension-gel coating may lack reproducibility, leading to significant variability between samples. This deviation suggests that the application of microneedles coated with the CLO-Sus gel may not be suitable for clinical use. In polymer-based formulations, a burst release may occur due to the delayed solidification of polymer precursors, leading to incomplete drug encapsulation. As a result, a fraction of the drug remains unencapsulated and is rapidly released in an initial burst [103,104]. A key challenge associated with rapid release is its unpredictability, even when a burst effect is beneficial (e.g., wound healing), and the extent of the release cannot be precisely controlled, posing potential pharmacological risks. It is important to highlight that drug release kinetics can be modulated through the strategic incorporation of swellable excipients [103]. Catellani et al. demonstrated that variability in release rates could be minimized by adding swellable and soluble polymers, such as hydroxypropyl methylcellulose (HPMC) or (poly(vinyl alcohol) (PVA), into the inert base matrix, resulting in a near-constant drug release profile [105]. Additionally, the incorporation of overcoat layers during the fabrication process has been shown to mitigate a burst release, primarily by adding external coating layers that do not contain the drug [103]. In the present study, all three coating layers contained clotrimazole. However, in the case of the CLO-EtOH gel, the presence of ethanol contributed to enhanced layer uniformity due to its wetting properties and reduced surface tension [106]. Considering the potential applications of gel-coated microneedles and future research directions, the presence of ethanol in the gel formulation may also enhance drug solubility within the *stratum corneum* lipids. This could lead to modifications in skin hydration, lipid extraction, increased lipid fluidity, and alterations in keratinized proteins, thereby facilitating improved clotrimazole penetration into the skin [107]. This suggests that microneedles coated with CLO-EtOH gel may be a more suitable candidate for potential clinical applications compared to those coated with CLO-Sus gel. Interestingly, DoC-shaped microneedles showed a two-stage release with a burst release of the drug observed between 240 and 360 or 480 min, depending on the gel type. This could be explained by the specific shape of the DoC. Part of the gel may have been deposited in the recess between the cones due to cohesive forces, resulting in a slower release followed by a subsequent rapid release. It is also worth noting that significant variations in the amount of CLO released from microneedles with the DoC shape were observed. Therefore, it can be suggested that the DoC shape may not be the most suitable choice when developing therapies for treating skin fungal infections. To date, the majority of studies investigating drug release from microneedle systems have relied on membrane-based methods. However, Larrañeta et al. proposed two alternative approaches for studying the release of methylene blue-loaded MNs [108]. The first involved inserting the system into Parafilm M^®^, followed by Franz cell testing, while the second utilized a hermetic “pouch” system, in which two sealed Parafilm^®^ M/MN units were immersed in a beaker containing 30 mL of PBS (pH 7.4). Both approaches demonstrated comparable results, releasing ~80 µg of dye over 360 min. Nevertheless, it is crucial to acknowledge that the results observed during *in vitro* studies should be validated through *ex vivo* experiments using skin, as well as *in vivo* studies on animal models, and further supported by clinical trials to ensure translational relevance.

The microbiological analysis confirmed the antifungal efficacy of CLO-coated microneedles against *Candida albicans* strains. Notably, the effect against the clinical origin strain was greater compared to the reference strain, a phenomenon frequently reported in the literature [109,110,111]. Variations in genome sequence or size within the same species can occur depending on whether the strain was isolated directly from an infection or maintained in a laboratory collection [109]. For instance, Alnuaimi et al. reported that *Candida* laboratory reference strains and clinical isolates form biofilms at different rates [110]. Furthermore, our findings suggest that the increased growth inhibition observed for MNs coated with CLO-EtOH gel may be related to the enhanced solubility of CLO in ethanol, facilitating improved drug diffusion. Nonetheless, MNs coated with the placebo-EtOH gel showed no inhibitory effects, indicating that ethanol alone did not influence microbial growth during the assay. This was attributed to the evaporation of volatile components, including ethanol, during the drying process. Consequently, in the case of placebo-EtOH gel coating, the residual ethanol content was minimal and insufficient to inhibit the growth of *Candida albicans*. Ethanol served a technological function in the manufacturing process, enabling the dissolution of the drug, which has very low solubility in water (0.49 μg/mL). Additionally, it facilitates the uniform distribution of the gel on the microneedle surface and enhances drug deposition, ensuring a more effective coating of the microneedles. Nevertheless, even a small amount of ethanol, when combined with a potent antifungal agent such as CLO, could lead to a potential synergistic effect. In addition to the improved diffusion of the drug in its dissolved form, this effect may also explain the significantly greater inhibition observed for microneedles coated with CLO-EtOH gel compared to those coated with CLO-Sus gel. Interestingly, after 48 h, differences in inhibition zones between CLO-EtOH and CLO-Sus gels were generally smaller for both strains, potentially due to delayed growth or improved visualization of partially inhibited microorganisms.

Microtox^®^ is a widely applied bioassay known for its rapid execution and relatively low cost. While it has been primarily utilized for assessing the toxicity of aquatic environments, sediments, and soil conditions, its application in evaluating the toxicity of drugs and materials has gained increasing attention [70]. The acute toxicity analysis confirmed that all tested microneedle formulations were non-toxic to bacteria, demonstrating the biocompatibility of the photopolymerizable resin used in their fabrication. The selection of non-toxic materials is crucial for microneedle production, particularly given their potential applications in topical drug delivery. The results suggest that the incorporation of clotrimazole has an impact on toxicity, as microneedles coated with either CLO-EtOH or CLO-Sus gels exhibited a slightly higher reduction in bioluminescence compared to placebo-coated or uncoated samples. Additionally, MN systems coated with the CLO-EtOH gel induced a greater bioluminescence decrease compared to those coated with the CLO-Sus gel, suggesting that the observed effect may result not only from the drug, but also from the presence of ethanol. However, all values remained below the established toxicity threshold. The presence of negative values may indicate a potential hormetic response induced by the tested formulations [71]. Previous studies have reported toxicity associated with resin compositions, demonstrating their effects on decreased bacterial bioluminescence [112]. Nevertheless, an in-depth cytotoxicity evaluation is required, preferably with additional studies using human cell lines, as they provide a more comprehensive evaluation of biocompatibility [113]. Moreover, *in vivo* skin compatibility tests could provide a broader insight into the safety and potential clinical application of 3D-printed microneedle systems. In the study presented by Bal et al., involving 18 volunteers, the *in vivo* skin compatibility of solid and hollow microneedles was evaluated [114]. It was observed that as the microneedle length increased, the values of transepidermal water loss (TEWL), blood flow, and skin redness also increased. Furthermore, hollow microneedles induced higher TEWL values than solid microneedles, while causing less skin irritation. However, in the case of all microneedles, irritation was minimal and lasted for less than two hours, which further supports the potential applicability of microneedle systems.

## 5. Conclusions

The study presented an optimized approach for the LCD-based 3D printing of microneedle (MN) systems, enabling the development of various geometries, including cone (shape A), double overlapping cone (DoC) (shape B), and pyramid with a hexagonal base (shape C). The research comprehensively addresses the adjustment of key printing parameters, such as layer thickness (0.03 mm), exposure time (7.5 s), and printing angle (shapes A and C: 45°, shape B: 60°), to ensure structural integrity, dimensional accuracy, and adequate mechanical strength of the 17-needle patches. The MN systems were coated with two types of hydrogels: ethanol-based (CLO-EtOH gel) and suspension-based (CLO-Sus gel), with microscopy analysis confirming that the EtOH gel formed a smoother and more uniform coating on the MN surface. The fabricated MN systems, characterized by sharp tips, successfully penetrated up to three layers of Parafilm^®^ M and perforated agarose gels, confirming their capability for skin penetration. Drug release studies revealed distinct release profiles depending on the coating formulation, with the Sus-gel exhibiting a higher cumulative release of CLO within the first 60 min compared to the EtOH gel. Additionally, the DoC-shaped MNs revealed a burst release phase at 240 min, potentially influenced by their complex design. Furthermore, CLO-coated MNs demonstrated antifungal activity against both reference and clinical origin strains of *Candida albicans*, with the reference strain exhibiting higher resistance to the drug. The MN systems coated with CLO-Sus gel exhibited slower diffusion, resulting in a smaller inhibition zone compared to those coated with CLO-EtOH gel. Lastly, an acute toxicity assay confirmed the biocompatibility of the developed MN systems, with no significant toxic effect observed.

Multiple studies suggest that MN-based drug delivery systems hold significant potential for treating various diseases, including fungal infections. However, several challenges remain, including the selection of microneedle types, optimal geometries, fabrication technologies, drug loading capacities, and safety considerations. Future studies should incorporate *ex vivo* penetration tests using full-thickness human skin and be further validated through *in vivo* experiments in animal models to ensure the translational relevance of research findings.

## Figures and Tables

**Table 1:** Comparison of key 3D printing parameters for SLA, DLP, LCD-based, and TPP 3D printing technology.

	SLA	DLP	LCD-Based	TPP
Light source	laser	light projector	array of light-emitting diodes (LEDs)	femtosecond laser pulses
Resolution	laser spot size: ~30–140 μm	pixel size: ~25–40 μm	pixel size: ~18–35 μm	laser spot size: ~100–200 nm
Speed (printing time)	14–50 mm/h (long)	20–140 mm/h (relatively quick)	20–80 mm/h (relatively quick)	~10 µm/s (long)
Printing material (light wavelength)	photosensitive resin (355–405 nm)	photosensitive resin (385–405 nm)	photosensitive resin (405 nm)	photosensitive resin (variable)
Cost	~2500–10,000 $	~15,000–30,000 $	~150–1000 $	from ~250,000 $
Post-processing	required	required	required	required

**Table 2:** Composition of the gels used in the tests [g] [60,61].

	CLO-EtOH	Placebo-EtOH	CLO-Sus	Placebo-Sus
Clotrimazole	0.50	-	0.50	-
Ethanol 96%	25.18	25.18	-	-
Glycerol	5.00	5.00	5.00	5.00
Carbopol^®^ EZ-3	0.50	0.50	0.50	0.50
Triisopropanolamine	0.75	0.75	0.75	0.75
Water	18.07	18.57	43.25	43.75

**Table 3:** Dimension (length and base width) of microneedle systems fabricated using 0.1, 0.05, or 0.03 mm layer thickness. Data are presented as mean ± SD (*n* = 3).

Shape A		Shape B	Shape C
Layer Thickness [mm]	Length [µm]	Length [µm]	Length [µm]
0.1	1479.47 ±24.48	1609.33 ±44.00	1494.83 ±8.12
0.05	1505.13 ±13.14	1639.40 ±32.28	1616.50 ±25.37
0.03	1636.80 ±33.57	1670.00 ±33.78	1546.00 ±34.70

**Table 4:** Dimension (length and base width) of microneedle systems fabricated using 1, 3, 5, 7.5, and 10 s of curing time. Data are presented as mean ± SD (*n* = 3).

	Shape A		Shape B		Shape C	
Curing Time [s]	Length [µm]	Base Width [µm]	Length [µm]	Base Width [µm]	Length [µm]	Base Width [µm]
1	failed to print					
3	1537.67 ±30.35	943.00 ±42.93	failed to print		1441.67 ±55.18	926.00 ±26.66
5	1606.33 ±70.81	1004.67 ±4.04	1670.00 ±33.78	789.67 ±9.61	1546.00 ±34.70	978.67 ±11.93
7.5	1670.33 ±20.21	1022.67 ±18.50	1742.67 ±19.60	802.33 ±9.29	1698.67 ±16.17	1012.00 ±14.80
10	1652.67 ±61.58	1009.67 ±36.00	1683.33 ±37.43	794.67 ±15.28	1605.33 ±50.00	1010.33 ±11.37

**Table 5:** The cumulative amount of drug extracted from each shape of microneedle systems. Data are presented as mean ± SD (*n* = 3).

	Drug Content/Microneedle System [µg]		Drug Content/Single Microneedle [µg]	
	CLO-EtOH	CLO-Sus	CLO-EtOH	CLO-Sus
Cone	168.60 ± 8.76	243.89 ± 15.81	9.92 ± 8.76	14.35 ± 15.81
DoC	166.36 ± 27.68	220.53 ± 9.42	9.79 ± 27.68	12.97 ± 9.42
Pyramid	174.51 ± 5.83	217.62 ± 8.56	10.27 ± 5.83	12.80 ± 8.56

## Data Availability

The original contributions presented in this study are included in the article. Further inquiries can be directed to the corresponding authors.
